# Urine tenofovir adherence testing: Perspectives of recently diagnosed South African adolescents and young adults with HIV accessing care via mobile HIV clinics

**DOI:** 10.1371/journal.pone.0318308

**Published:** 2025-01-31

**Authors:** Michalina A. Montaño, Siyaxolisa Sindelo, Amanda Fata, Elzette Rousseau, Linda-Gail Bekker, Ingrid T. Katz, Paul K. Drain

**Affiliations:** 1 Department of Global Health, University of Washington, Seattle, Washington, United States of America; 2 Fred Hutchinson Cancer Center, Seattle, Washington, United States of America; 3 Desmond Tutu HIV Centre, University of Cape Town, Cape Town, South Africa; 4 Department of Medicine, Brigham and Women’s Hospital, Boston, Massachusetts, United States of America; 5 Department of Medicine, Harvard Medical School, Boston, Massachusetts, United States of America; 6 Department of Epidemiology, University of Washington, Seattle, Washington, United States of America; Stellenbosch University, SOUTH AFRICA

## Abstract

**Background:**

Adolescents and young adults (AYA) living with HIV face several challenges to engaging in HIV care, which can impact adherence to antiretroviral therapy (ART). Point-of-care (POC) diagnostics that detect tenofovir in urine may be a useful tool to support ART adherence, but perspectives from AYA in South Africa have not been explored.

**Methods:**

We conducted in-depth interviews (IDIs) among young people (age 18–24) newly diagnosed with HIV in Cape Town, and a focus group discussion (FGD) with HIV care providers to understand their perspectives regarding the use of POC urine tenofovir testing to support ART adherence. Transcripts were analyzed using Dedoose, with an iterative thematic approach.

**Results:**

Transcripts from 8 IDI participants and 8 FGD participants were included in the analysis. Major themes identified during analysis related to beliefs about POC urine adherence testing and recommendations for future clinical implementation. Most IDI participants indicated they would want to use the tests if clinically available, and both IDI and FGD participants believed the tests would be helpful to clinicians. Participants believed the tests could motivate people to take their ART regularly, either by reassuring them ART was present in their bodies, or to avoid the negative consequences of being found to be non-adherent. Drawbacks of POC adherence testing identified by respondents included not wanting to be caught skipping ART doses, concerns about privacy, how the test results would be explained, and adding to the amount of testing required for HIV clinical care.

**Conclusions:**

AYA living with HIV in South Africa had favorable views toward POC tenofovir adherence testing and felt utilizing these tests in HIV clinical care would motivate people to remain adherent to ART.

## Introduction

Adolescents and young adults (AYA) aged 15–24 years comprise a third of new HIV infections in Eastern and Southern Africa, with new infections three times higher among AYA girls and women than males in the same age group [1]. Among people with HIV in this region, AYA is the only age group for whom HIV-related mortality is not declining [2]. In the Republic of South Africa (RSA), HIV care engagement among AYA lags behind other groups, with a 2017 national population-based survey finding only 40% of 15–24-year-olds with HIV were on treatment, and 48% of 15–24-year-olds were virally suppressed [3]. Longitudinal data from South Africans with HIV in this age group show inconsistent treatment adherence and low rates of sustained adherence over time [4].

The RSA National Department of Health recommends viral load testing at 3 and 10 months after antiretroviral therapy (ART) initiation, and every 12 months thereafter, to monitor treatment among people with HIV [5]. While this approach serves as a proxy for adherence monitoring, decreased or inconsistent adherence can take weeks or months to result in loss of virologic control [6,7]. Moreover, viral load monitoring may not detect early problems with adherence, leading to delays or missed opportunities to support better adherence proximal to ART initiation. Offering additional adherence support to AYA who need it requires identification of those who are struggling to maintain good ART adherence, but accurate measurement of ART adherence is a challenge [8]. Most clinical settings rely on self-reported measures and pill counts to assess adherence, but these techniques are often inconsistent, imprecise, and subject to social desirability bias [8,9]. Without accurate measurement of adherence in HIV care settings, it is difficult to ascertain who needs adherence support or additional intervention to remain engaged in treatment.

Objective measures of adherence have the potential to improve ART adherence monitoring by improving clinical assessment of adherence, and providing actionable information about adherence more rapidly than viral load monitoring [9]. Advances in point-of-care (POC) diagnostics have recently led to the development of lateral flow assays to detect tenofovir in urine. These highly accurate qualitative POC tests are more inexpensive and straightforward to use for measurement of recent ART adherence than other objective measures such as serum drug levels or detection of ART in hair or dried blood spots [10,11]. Although not currently in use in clinical settings in South Africa, they may be a practical tool for providing prompt information about adherence that can be used to identify people with HIV who need additional adherence support. POC urine tenofovir testing has been found to be acceptable among adults with HIV and female PrEP users, but the acceptability of using these tests to support adherence remains understudied, particularly among AYA [12,13]. To address this gap, we conducted a qualitative study to understand the perspectives of South African AYA and healthcare providers in a mobile clinic environment regarding the use of POC urine tenofovir testing to support ART adherence for newly diagnosed AYA.

## Methods

### Standing tall

The qualitative study was performed as a sub-study within *Standing Tall*, a pilot randomized controlled trial (ClinicalTrials.gov ID NCT04568460) designed to address multi-factorial barriers to ART uptake among 100 adolescents and young adults using 1) a socio-behavioral group intervention, 2) social support, and 3) provision of immediate ART and refills [14].

### Study design and population

We conducted interviews with South African AYA recently diagnosed with HIV, and a focus group discussion with healthcare providers involved in the delivery of HIV care. For the interviews, we utilized a convenience sampling approach, recruiting participants who had enrolled in the parent study, using the same inclusion and exclusion criteria. Individuals could be included if they 1) were between the ages of 18 and 24, 2) presented to Desmond Tutu Health Foundation (DTHF) mobile clinic vans in Cape Town, 3) had been diagnosed with HIV in the last four weeks and were ART-naïve at time of enrollment, 4) were English or isiXhosa speakers, and 5) provided informed consent. Individuals were excluded if they were pregnant, tested positive for tuberculosis, or were unable to understand the process of informed consent. For the focus group discussion, we recruited participants from DTHF mobile clinic staff involved in the provision of HIV-related clinical care. DTHF mobile clinics do not employ physicians, so this cadre of healthcare workers was not included in the focus group discussion.

### Data collection

Interview and focus group discussion guides were developed by the study team, with the goal of understanding the perspectives of South African young people and HIV care providers regarding the use of POC urine tenofovir testing to monitor ART adherence. Interviews and focus group discussion were conducted in person by a single trained interviewer (SS), a DTHF researcher known to participants and healthcare staff. Each interview participant spoke in a one-on-one conversation with the interviewer. Interview participants were recruited via the parent study by the interviewer, who explained the interview process and arranged a time and place to conduct each interview. Prior to interviews, the interviewer showed participants the POC urine test kit and explained to them how it worked. Participants were shown the SureQuick Rapid Tenofovir Adherence Test (OraSure Technologies Inc., USA), and told this qualitative test detects tenofovir intake during the prior 48 hours [11]. Due to lack of regulatory approval to disclose test results, the interviewer did not perform the test or disclose results to participants. During interviews, participants were asked about their understanding of ART adherence and the advice they had received from healthcare providers about ART, their beliefs about urine adherence testing, and recommendations for future implementation in clinical settings. Interview participants were also asked how they would respond if the test result was discrepant from their self-perceived adherence, for example if the test showed no tenofovir in their urine but they perceived their adherence to be high. During the focus group discussion, healthcare providers were asked about the benefits and drawbacks of urine adherence testing and their recommendations for future implementation. Both the interviews and the focus group discussion were conducted in isiXhosa and were audio recorded. Audio files were transcribed and translated into English for analysis.

All participants provided written informed consent. This study was approved by the University of Cape Town Human Research Ethics Committee (IRB00001938) and the Mass General Brigham Institutional Review Board (2019P002671).

### Analysis

The interviews and focus group discussion were analyzed using an iterative thematic approach. Transcripts were analyzed in English using Dedoose software (Los Angeles, CA). One investigator (MM) drafted a preliminary codebook based on topics addressed in the interview guide. Three members of the study team (MM, SS, AF) pilot coded one of the transcripts individually and then met to discuss findings, reconcile discrepancies, and finalize the codebook. Each researcher coded 4 or 5 interviews. All interview transcripts were double-coded, and all three researchers coded the focus group discussion transcript. Each discrepancy in coding was discussed and resolved in group discussion among all three coders. The team met again to discuss major themes which arose from the text and came to an agreement on the most relevant excerpts to include in the analysis. Excerpts were selected based on how clearly they elucidated each theme.

### Positionality

The lead author (MM) is a white, American researcher at a US academic institution. As a white person conducting research among young, Black, South African people with HIV, this author recognizes the limitations of her personal experience and the potential for bias in interpretation and presentation of findings. In this study, it was essential to partner with South African researchers at DTHF (SS, ER, LGB) to assist with development of focus group discussion and interview guides, to lead interviews and focus group facilitation, and to collaborate on analysis and interpretation of results. The study described here would not have been possible without the guidance and contributions of the DTHF research team.

## Results

Interviews were conducted with 10 participants between November 2020 and September 2021. Two interview audio recordings had playback issues and were not included in the analysis. The coding team determined saturation of responses had been reached within the 8 remaining interviews during analysis. Most participants were female, and in their early 20s (Table 1). Participants engaged in interviews for an average of 15 minutes. The focus group discussion included 8 healthcare workers – two nurses, three counsellors, a retention officer, a research administrator, and a facilitator – and lasted one hour. Major themes identified during analysis related to beliefs about urine POC adherence testing, reactions to discrepant or unexpected results, and recommendations for future POC urine test implementation.

**Table 1:** Demographic characteristics of interview participants (N =  8).

Gender	
Female	7 (87.5)
Male	1 (12.5)
Age; median (range)	21 (19–24)

### Beliefs about POC urine adherence testing

Interview participants were asked about the benefits and drawbacks of using POC urine tests to assess ART adherence. All interview participants understood the purpose of the test, although one initially conflated it with viral load testing. When asked about drawbacks or potential reasons a person might not want to use the tests, the most widely listed reason was that people who were not taking their ART regularly or had recently skipped doses would not want it known they were not adhering to their ART regimen.

*“It’s the fact that it will tell the truth… In that [if] I am no longer using my ARVs and that’s why. It’s because people don’t want to take their ARVs and people just go to the clinic to get them for the sake of their families but then throw them away in the bin and not use them at all and when they do that, that’s when their soldiers in their bodies start becoming weak and decrease and then they die from HIV which is something that shouldn’t kill them at all.” – Female participant, age 24*

Other drawbacks included concerns about confidentiality, what would happen to urine samples, confusing explanation of the test, irritation about frequency of testing required for HIV clinical care, and a desire for the test to detect tenofovir over a longer period of time.

When asked about the benefits or reasons a person might want to use the tests, most participants indicated they would want to use the tests if they were available during their HIV care visits. Participants largely thought that having the tests available as a regular part of clinical care would motivate people to continue taking their ART regularly. Participants thought the tests would promote good ART adherence for two main reasons. First, participants believed people would be more likely to take their ART if their tenofovir levels were regularly checked to avoid negative consequences of being found to be non-adherent. Second, participants felt that seeing a positive result showing the presence of ART in their urine would alleviate anxiety about whether their ART was working by reassuring them that it was present in their bodies, which would motivate them to continue taking their pills.

*“A person might even be motivated to continue taking it if they see that they’re being found in their body – and see that they’re really working… Cos other people say they don’t see the reason of even taking the pills, cos they don’t know whether they’re working or not.” – Female participant, age 20*

*“It will force you to keep taking your ARVs… Cos it will show at the clinic every month to see what is happening and how ARVs you have. And if you’re taking them the right way.” – Female participant, age 22*

In addition, most interview participants believed it would be helpful for healthcare providers to know whether patients were adherent to their ART.

*“I think it would be very useful … How they do things here is that they will give you your pills, and instruct you to come back after a few days to check if you are really taking the pills. So, they don’t really have the proof that I am really taking my pills, and I could go there without ever taking them. When they call you to come back, they count them to check if you’re really taking them. But, that doesn’t help cos I could just empty the package, and claim that I am taking them.” – Female participant, age 22*

Focus group participants were also queried about the advantages and disadvantages of using POC urine tests to assess ART adherence for clients with HIV. Similar to interview participants, focus group participants believed using the tests in clinical practice would have a positive influence on adherence by motivating clients to take their ART.

*“I think it’s good that there’s a test like this and it will also encourage them to use their tablets because they know that they will be tested and there’s no use of lying.” – Focus Group Participant*

Focus group participants also pointed out these tests could provide useful oversight for healthcare providers, and that they might be more accurate than self-report or pill counting. However, others believed the two-day window might not accurately reflect long-term adherence.

*“I feel like 2 days is way too short to determine whether a person is taking their ARVs or not… Let’s say that a person hasn’t been taking their pills for the rest of the month and they know that, ‘On Friday, I’m going to the truck,’ they can just take that 2 days only.” – Focus Group Participant*

### Discrepant or unexpected results

Interview participants were shown the urine tenofovir test kits during the interview, but the tests were not used and participants were not given test results. They were asked about how they might feel if they received a result they weren’t expecting, in particular, if the test showed no tenofovir in their urine but they thought they had been taking their ART regularly. The responses to this question varied widely. One participant felt that getting a discrepant test result could be demotivating and discourage adherence if coupled with a negative reaction from a healthcare provider.

*“I would feel bad because I know that I am taking them each and every day. Because if it’s gonna say that I am not taking them the right way when I am, for instance… there’s this other sister (nurse) of ours where I go who deals with HIV and she’s very rude so if this test ever came and said that you have not been taking these pills for the past few days even though you’ve been taking them, I don’t know… It’s things like that that make you feel small and never want to take the treatment ever again.” – Female participant, age 24*

Another responded that they were confident they were taking their ART correctly, and a negative test result would undermine their confidence in the ART pills or the test itself.

*“Like, it shows that they are not in my body?... It means there’s a problem with the pills that I am taking. Maybe… I don’t know what to say… [If] they’re not in your body, and you’ve been taking them. [I would think] that there’s something wrong with the test.” – Female participant, age 20*

Other participants indicated they would be concerned they were taking their ART incorrectly or would want more information to help explain the test results.

*“I would want to… I would want to fix my ways cos maybe I am taking them incorrectly… I would want to know more.” – Female participant, age 22*

### Recommendations for future implementation

Interview participants had several recommendations for potential future implementation of the POC urine tenofovir tests in HIV care settings. These recommendations fell into two main categories: recommendations for the test device itself, and recommendations for its integration into HIV care. Regarding the test itself, participants expressed a desire for a test that would give them other results as well, such as CD4 count and viral load). One participant additionally felt it would be more useful if the tenofovir detection window was longer.

*“Another thing I would like the device to tell me is if my body is performing well or what like for instance, with the CD4 count, if it’s going up or down and stuff… So, if I know that I’m not taking it well and it shows me that my CD4 count is going down, that’s where I’ll be motivated to take my pills up until it’s not going down cos if it goes lower there will be problems.” – Female participant, age 24*

Regarding integration of the test into HIV care, participants were asked specific questions about their preferences for the timing of the test during HIV care visits and who would administer the tests and disclose results. Four of the eight interview participants preferred to have the test done at the beginning of their visit, three had no preference about the timing of the test, and one participant expressed a desire to have the test done at the end of their clinical visit. Five participants had no preference regarding who would administer the test and disclose results, two expressed a preference for a doctor to explain the test and give them results over a nurse or counsellor, and one participant said they would want a friend to disclose their results rather than a healthcare provider.

Focus group participants discussed several recommendations for implementing POC urine adherence testing in healthcare settings, including how the test might be best integrated into HIV care. Participants highlighted the importance of transparency in how the test would be explained, how clients would be notified that the test would be performed, and how test results would be communicated. In addition, they thought contextualizing the test with other HIV-related testing and health indicators would be necessary.

*“It would be the same as explaining the CD4 count. In that, ‘if you take your ARVs, your immune system gets stronger, and the viral load is suppressed. You were not on ARVs, right? And, we’ve just discovered that you now have HIV, you will start your medication. Now, we have this device that will look to see if the medication is being received by your body.’” – Focus Group Participant*

Most focus group participants agreed that counsellors should be involved in disclosing test results due to their training in confidentiality and disclosing test results, and their experience in having potentially difficult conversations. They pointed out the importance of transparency in results disclosure, and that additional conversations with clients would be necessary to understand the context of their adherence behavior, particularly in the case of negative test results.

*“At the end of the day, that is part of adherence. And, the counsellor and the nurse… I think they are the ones most educated on that. Of which, I think, especially as counsellors, this is our job… counsellors are the ones who have to teach them again how to maintain a level of taking pills.” – Focus Group Participant*

## Discussion

Our study exploring AYA and healthcare provider perspectives on POC urine tenofovir adherence testing revealed most AYA participants had favorable views toward POC adherence testing, and indicated they would want to use the tests if available. Most participants believed having POC urine adherence tests utilized during HIV care would motivate people to remain adherent to their ART, either to reassure them ART was present in their bodies, or to avoid non-adherence being discovered. Some stated a test result showing the presence of ART in their urine would reassure them if they had uncertainty about whether the medication was effective. Others pointed out that the anticipation that ART adherence would be monitored by a clinical test might motivate some people to remain adherent. Healthcare worker participants agreed that the test would motivate people with HIV to remain adherent to ART if used in clinical settings.

This is the first qualitative study to evaluate AYA perspectives on utilizing POC urine tenofovir assays to measure ART adherence, although this topic has been explored among older adults with HIV. Our findings are similar to interview responses from adults with HIV in Lesotho, many of whom also reported that having these tests implemented in clinical practice would motivate them to remain adherent to their ART between clinical visits [12]. Another qualitative study conducted among older adults with HIV in Cape Town similarly found that POC urine tests were generally acceptable and might encourage good adherence [15]. Additionally, prior studies from the region have identified negative perceptions about ART efficacy as a barrier to adherence, which is reflected in our finding that test results showing the presence of ART would be reassuring to participants uncertain about medication efficacy [16,17]. While there is limited data on the impact of biofeedback on ART adherence, a recent pilot randomized trial conducted in RSA found that urine tenofovir testing with biofeedback counseling combined with HIV self-testing improved PrEP adherence among postpartum women [18]. Studies from other countries similarly suggest that drug level monitoring among PrEP users is acceptable and may improve adherence [19,20]. Our findings suggest that the same may be true for AYA with HIV.

Participants had a wide variety of responses to questions about receiving discrepant results, ranging from assuming the test would be correct and wanting to know what they should change about their pill-taking, to questioning whether a result showing no presence of ART might be due to a problem with the test or their ART pills. Since participants were not given POC urine test results during the interviews, these questions were hypothetical, and participants may have had difficulty predicting how they would react to unexpected results without actually experiencing disclosure of results. However, the range of hypothetical reactions illustrates the need for a clear explanation of the purpose of the tests, providing information about POC adherence testing within the context of HIV clinical care and other routinely administered tests, and clear protocols for disclosing results, answering questions, and referrals for adherence counselling if indicated. This is supported by recommendations of healthcare workers participating in the focus group discussion. These participants highlighted the importance of clearly explaining the purpose and context of the POC urine tests and utilizing the skills of trained counsellors to disclose results and incorporate this process into adherence support.

Limitations of our study include that the participants were receiving HIV care through the organization conducting the interviews and may have felt uncomfortable disclosing negative perspectives on the POC tests. Despite the limited sample size, which may limit generalizability, we reached saturation, and interview findings were further substantiated by findings from the focus group discussion. AYA participants interviewed for this study were engaged in HIV care at the DTHF mobile clinics. While we reached saturation in this population, their views on tenofovir adherence testing may not be representative of AYA with HIV not engaged in care, or those accessing care via other means. Exploring the opinions of other AYA groups will be important for guiding broader implementation of POC ART adherence testing in other HIV care settings. Additionally, while participants were able to view the POC tests, tests were not performed, and test results were not disclosed as part of the interview process. Participant perceptions presented in this study may differ from those of clients experiencing these tests as part of HIV clinical care. A subsequent series of interviews has been planned to include performance of the tests and disclosure of results in order to explore experiences related to receiving results in more depth. Finally, physicians were not included in the FGD, although many IDI participants indicated they would prefer to receive adherence results from physicians. Including this cadre of healthcare worker in future research on this topic will be necessary to understand the breadth of healthcare worker perspectives.

In conclusion, POC urine tenofovir adherence tests are promising new tools for supporting ART adherence among AYA with HIV, who often struggle to adhere to ART regimens more than other age groups. AYA participants in this study had overall favorable opinions about POC adherence testing and felt utilizing these tests in HIV clinical care would motivate people with HIV to remain adherent to ART. Healthcare workers agreed these tests could have a positive influence on ART adherence for this population. Studies are needed to measure effectiveness of these tests for supporting ART adherence among AYA with HIV, and establish best practices for implementing POC adherence testing, disclosing results, and integrating these tests into existing processes for adherence support.

## Supporting information

S1 File Checklist. (DOCX)
